# A brief review of the VI-RADS classification for bladder tumors on MRI (and a call for increased interface, consistent communication and more joined studies by the radiological and urological communities)

**DOI:** 10.1590/S1677-5538.IBJU.2021.0560.1

**Published:** 2022-07-19

**Authors:** Ronaldo Hueb Baroni

**Affiliations:** 1 Departamento de Radiologia Faculdade Israelita de Ciências da Saúde Albert Einstein São Paulo SP Brasil Departamento de Radiologia, Faculdade Israelita de Ciências da Saúde Albert Einstein, São Paulo, SP, Brasil; 2 Departamento de Imagem Hospital Israelita Albert Einstein São Paulo SP Brasil Departamento de Imagem, Hospital Israelita Albert Einstein São Paulo, SP, Brasil

## COMMENT

In 1993 the American College of Radiology (ACR) proposed a new classification for breast tumor evaluation and reporting on mammography, called BI-RADS (Breast Imaging Reporting and Data System), a standardized lexicon, which was developed on the back of the established 5-tier ACR system (a Likert scale) ( 1 ). The aim of BI-RADS was to improve distinction between benign and malignant diseases, to remove ambiguity from radiology reports, permit automated auditing of data and improve clinical interface with the referring physicians. Nowadays, there are more than twenty RADS available for the radiological evaluation of many diseases and organs, nine of then developed under the ACR criteria and supervision, including two that are more widely used and well know: LI-RADS (liver cancer) and PI-RADS (prostate cancer) ( 2 ).

In 2018, a multidisciplinary group of radiologists, urologists, pathologists and radiation oncologists developed and published a new scoring system called VI-RADS (Vesical Imaging-Reporting and Data System), focused on the local staging of bladder cancer on MRI, including the standardization of MRI protocols and the proposal of a structured reporting system to improve communication between referring physicians and radiologists ( 3 ). The main goal of the proposed system was to overcome, through a non-invasive imaging method, the risks and limitations of transurethral resection of bladder tumor (TURBT), such as bladder perforation and under/overstaging. The system relies on a 5-point scale (VI-RADS 1 to VI-RADS 5), using multiparametric MRI (that includes high-resolution T2-weighted, diffusion-weighted, and dynamic contrast-enhanced imaging), to stratify the risk of invasion of the muscular layer of the bladder wall in a previously detected lesion. VI-RADS rapidly gained acceptance by the radiological and urological communities, and many multicentric studies were published since then confirming that the system has excellent interobserver agreement and accuracy for local staging. Those studies include two systematic reviews and meta-analysis, published in 2020 by Woo et al ( 4 ) and Luo et al ( 5 ), that found similar AUC accuracies for local staging of bladder cancer using VI-RADS (between 0,92 and 0,94) Another systematic review and meta-analysis, published in 2022 by Del Giudice et al. ( 6 ), focused on inter-reader reproducibility and found a Cohen’s Kappa of 0,83.

The article from Nicola et al ( 7 ), gives a very comprehensive and step-by-step review of the many aspects of VI-RADS, targeting the urological community. It is of utmost importance that urologists (as well as clinical oncologists and radiation oncologists) become familiar with the applications, limitations and basis of imaging interpretation of the system. Being a relatively young classification (as compared to BI-RADS, LI-RADS and PI-RADS), VI-RADS demands more prospective and multicentric studies to further validate its already excellent results.

I believe that one of the major strengths of VI-RADS relies on the fact that it was developed from the very beginning with the inputs of all involved “stakeholders” (radiologists, urologists, pathologists and radiation oncologists). This should be a must for all studies that intend to standardize how we perform, read and report an imaging exam. The PI-RADS steering committee consider the PI-RADS classification (now in version 2.1) as a “living document”, since continuous improvements will certainly occur and be incorporated in the newer versions. I am positive that the same idea is valid for VI-RADS. Let’s work together to make it even better and more widely used.
